# Association between eating behaviour and diet quality: eating alone vs. eating with others

**DOI:** 10.1186/s12937-018-0424-0

**Published:** 2018-12-19

**Authors:** Wonjeong Chae, Yeong Jun Ju, Jaeyong Shin, Sung-In Jang, Eun-Cheol Park

**Affiliations:** 1 Department of Public Health, College of Medicine, Yonsei University, Seoul, Republic of Korea; 2 Institute of Health Services Research, Yonsei University, 50 Yonsei-ro, Seodaemun-gu, Seoul, 120-752 Republic of Korea; 3 Department of Preventive Medicine, College of Medicine, Yonsei University, 50 Yonsei-ro, Seodaemun-gu, Seoul, 120-752 Republic of Korea

**Keywords:** MAR, NAR, Eating alone, Diet quality, Socioeconomic status

## Abstract

**Background:**

To discover the association between eating alone and diet quality among Korean adults who eat alone measured by the mean adequacy ratio (MAR),

**Methods:**

The cross-sectional study in diet quality which was measured by nutrient intakes, indicated as MAR and nutrient adequacy ratio (NAR) with the Korean National Health and Nutrition Examination Survey (KNHANES) VI 2013–2015 data. Study population was 8523 Korean adults. Multiple linear regression was performed to identify the association between eating behaviour and MAR and further study analysed how socioeconomic factors influence the diet quality of those who eat alone.

**Results:**

We found that the diet quality of people who eat alone was lower than that of people who eat together in both male (β: − 0.110, *p* = 0.002) and female participants (β: − 0.069, *p* = 0.005). Among who eats alone, the socioeconomic factors that negatively influenced MAR with the living arrangement, education level, income levels, and various occupation classifications.

**Conclusions:**

People who eat alone have nutrition intake below the recommended amount. This could lead to serious health problems not only to those who are socially disadvantaged but also those who are in a higher social stratum. Policy-makers should develop strategies to enhance diet quality to prevent potential risk factors.

**Electronic supplementary material:**

The online version of this article (10.1186/s12937-018-0424-0) contains supplementary material, which is available to authorized users.

## Background

Food intake is an essential factor related to health status [1–4]. Adequate nutrients must be consumed through diet in order to survive. These days, people who care about what they eat think about how it will affect their well-being and lifestyle rather than how it can help to survive day-to-day life [5, 6]. When we observe how food is produced and consumed, there is no significant difference compared to the past [7]. However, the range of nutrients consumed has expanded widely and eating behaviours have changed with economic growth. Diet quality is described in nutritional epidemiology literature in a variety of ways, including a healthy diet, balanced diet, nutritious foods, functional foods, and nutrient-rich diet [8]. Those terms point to the bottom-line idea of achieving an optimal level of health via a balanced nutrient intake.

Academic research to investigate the relationship between diet quality and diseases are increasing. There are studies that show effects of healthy eating and a reduction in risk for chronic diseases [1, 9, 10] and mortality [9, 11, 12]. Studies among elderly Japanese population demonstrated that solitary eating negatively influenced meal quality, which is consistent with our theory [10, 12, 13]. Eating alone discourages people from having a well-balanced meal. Some even see eating alone as an efficient way of having a meal, and as a new trend is being adopted, we might have to face a socially isolated generation [13, 14]. Poor eating quality will become a new public health issue. Eating alone can cause vulnerability in nutrition among elderly population [15] yet commensality can influence positively to elder population [16]. In contrast, eating together, such as in a family, promotes the healthy eating habits to lead a healthy lifestyle [17]. Also the study on middle and high school students on the frequency of family meals, breakfast and dinner, shows the students who had a higher frequency of family meals had better dietary intake and quality than who did not have family meals [18].

With an increase in the interest in diet quality, eating behaviour has also evolved in modern society. The traditional definition of a meal includes companions such as family or friends [13]. The new form of living style brought the concept of eating alone (solitary eating), and it became a new trend in some generations. Selecting foods for a day is easily influenced by the social environment and eating together or alone plays a big role in that decision [2, 13, 19, 20]. When people plan to eat alone, the meal will not take long; usually they pick a simple and quick meal rather than a nutritionally balanced meal [13, 20, 21]. Regardless of this new trend, solitary eating causes dietary problems such as modern malnutrition [2, 11]. For people who eat alone, it is hard to consume adequate nutrients, especially for micronutrients due to the limited consumption of fruits and vegetables [2]. In fact, it leads not only to modern malnutrition, it could also cause clinical malnutrition due to insufficient nutrient intake [11]. Eating alone puts people at risk of potential illness, as has been reported in earlier studies [11, 12].

Previous studies were conducted to search for causative factors of illness due to nutrition. There are studies that found health status resulted from imbalanced nutrition [12, 22]. Also, some studies assessed the impact of socioeconomic status on nutritional health and eating patterns [20, 23]. Research on eating alone and quality of meals is comparatively new yet expected to increase in our society. By looking at the increasing prevalence of the elderly population and one-person households [24] and rapid changes in environmental factors [2, 25], we expect to see an increase in the prevalence of people who eat alone. Our study aims to evaluate the association between diet quality of the modern Korean adult population based on the eating behaviour and the socioeconomic factors that influence their diet quality.

## Methods

### Study subjects

This study was conducted with data from the sixth Korea National Health and Nutrition Examination Survey (KNHANES VI-3) by the Korea Centers for Disease Control and Prevention, which contains survey data for the cross-sectional study from the health interview survey, the health examination survey, and the nutrition survey from 2013 through 2015. The 2015 Dietary Reference Intakes for Koreans by the Korean Nutrition Society and Ministry of Health and Welfare, Korea were used to reference data for the recommended nutrient intakes [26]. The final population for this study is 3365 men and 5158 women who are age 19 to 64 years old.

### Dependent variable

The dependent variable of mean adequacy ratio (MAR) was used to measure the quality of the meal. In order to compute MAR, the nutrient adequacy ratio (NAR) of each nutrient was calculated by the formula below. In 1972, Madden and Yoder [27] first used MAR and NAR to evaluate food stamp efficiency and commodity distribution. NAR shows the ratio of each nutrient intake relative to the recommended dietary intake or recommended dietary allowance [27, 28]. For this study, we applied the Dietary Reference Intakes for Korean 2015 as the reference value of RDA and collected nutrients are protein, calcium, phosphate, iron, vitamin A, vitamin B1, vitamin B2, Niacin, and Vitamin C through the nutrition survey for assessment. The formulae for MAR and NAR are below [22]:

$$ \mathrm{MAR}=\mathrm{Sum}\ \mathrm{of}\ \mathrm{NAR}\ \mathrm{of}\ \mathrm{nutrients}/\mathrm{the}\ \mathrm{number}\ \mathrm{of}\ \mathrm{nutrients} $$

$$ \mathrm{NAR}=\mathrm{Nutrient}\ \mathrm{intake}/\mathrm{recommended}\ \mathrm{nutrient}\ \mathrm{intake} $$

NAR, which is obtained from the recommended nutrient intake, refers to sex and age-specific 0references for daily intakes. A NAR close to 1 indicates the consumed meal is near the recommended amount of that specific nutrient for the day; when it is > 1, it means the consumed meal exceeded the recommended amount of the nutrient [22, 27]. This study included various foods and beverages since both of them could be significant sources of nutrients.

### Independent variable of main interest

The variable of main interest was eating behaviour, which represents whether they have company during the meal. The variable was described using categorical data based on participants responding in “yes” or “no” format. The questions were asked by each meal, breakfast, lunch, and dinner. The final grouping was designed after considering the frequency of the meal and the existence of company during the meal (Box 1). The population was separated by gender, and then categorized them into three groups: ‘alone’ for those who ate all meals alone, ‘some together’ for those who ate some meals with others, and ‘together’ for those who ate with others for every meal.

### Covariates

This study included demographic, socioeconomic, and health behaviour factors as covariates. Demographic factors used to assess general characteristics of the study population included living arrangement, age group, and residential area. The socioeconomic factors reviewed were marital status, education level, income level, and occupation status in four categories (white collar: administrative and management role, blue collar: manual labour industry and pick collar: service industry). For the health behaviour factors, body mass index (BMI: underweight: BMI < 18.5, normal: 18.5 ≤ BMI < 25.0, and overweight: BMI > 25.0) weight changes in the past 1 year, alcohol consumption, smoking status, and stress level were assessed. We also added nutrition behaviour-related factors such as nutritional education, nutrition supplement intake more than 2 weeks per year, and nutritional fact usage to evaluate whether or not there are associations with MAR.

### Statistical analysis

To compare the proportion of variables by MAR, independent t-test and ANOVA were used and a univariate analysis was performed. Multiple linear regression analysis was used to determine an association between MAR and eating behaviour using a generalized linear model (GLM). We performed further analysis to discover an association between socioeconomic status and eating behaviour with diet quality measured by MAR.

## Results

The characteristics of the study population (*N* = 8523) are presented in Table 1. The participants were grouped by sex; there were 3365 (39.48%) men and 5158 (60.52%) women. The mean MAR for male and female populations was the same at 1.03 ± 0.48. Participants were also grouped into three categories based on their eating behaviour. In the male group, 256 (7.61%) ate every meal alone, 1199 (35.63%) ate some meals together, and 1910 (56.76%) ate every meal together. In the female group, 502 (9.73%) ate very meal alone, 2255 (43.72%) ate some meals together, and 2401 (46.55%) ate every meal together. Male participants’ MAR was not influenced by whether or not they were living together, while female participants’ MAR was influenced by their living arrangement (*p* < 0.001) (Table 1).

**Table 1:** General characteristics of the study population

Variables	General characteristics									
	Male					Female				
	Subject		MAR			Subject		MAR		
	N	%	MEAN	SD	*p*-value	N	%	MEAN	SD	*p*-value
Eating Type					<.001					<.001
Alone	256	7.61	1.033	0.483		502	9.73	1.035	0.476	
Some Together	1199	35.63	1.187	0.497		2255	43.72	1.143	0.473	
Together	1910	56.76	1.200	0.505		2401	46.55	1.148	0.472	
One person household					0.103					<.001
Yes	245	7.28	1.133	0.563		283	5.49	1.006	0.471	
No	3120	92.72	1.187	0.497		4875	94.51	1.142	0.473	
Age Group					<.001					<.001
19–29	614	18.25	1.292	0.553		764	14.81	1.125	0.499	
30–39	715	21.25	1.313	0.539		1120	21.71	1.186	0.491	
40–49	796	23.66	1.187	0.491		1293	25.07	1.115	0.448	
50–59	832	24.73	1.075	0.438		1370	26.56	1.139	0.466	
60 above	408	12.12	1.002	0.385		611	11.85	1.085	0.474	
Residential Area					0.064					0.370
Urban	2771	82.35	1.190	0.507		4364	84.61	1.137	0.474	
Rural	594	17.65	1.148	0.476		794	15.39	1.121	0.474	
BMI					0.013					0.901
Underweight	71	2.11	1.126	0.475		314	6.09	1.126	0.509	
Overweight	1341	39.85	1.213	0.507		1335	25.88	1.132	0.494	
Normal	1953	58.04	1.164	0.499		3509	68.03	1.137	0.463	
Weight Changes					<.001					<.001
Same	464	13.79	1.137	0.519		616	11.94	1.071	0.462	
Decreased	726	21.58	1.259	0.522		1554	30.13	1.167	0.512	
Increased	2175	64.64	1.167	0.489		2988	57.93	1.131	0.454	
Marital Status					<.001					<.001
Married	2365	70.28	1.170	0.481		3813	73.92	1.151	0.461	
Separated	144	4.28	0.936	0.409		504	9.77	1.023	0.462	
Single	856	25.44	1.260	0.556		841	16.30	1.127	0.529	
Education Level					<.001					<.001
Primary	247	7.34	0.950	0.431		654	12.68	0.991	0.431	
Secondary	273	8.11	1.047	0.454		532	10.31	1.093	0.471	
Upper Secondary	1355	40.27	1.213	0.521		2000	38.77	1.149	0.476	
Tertiary	1490	44.28	1.219	0.489		1972	38.23	1.180	0.476	
Income Level					<.001					<.001
Lowest	776	23.06	1.123	0.527		1243	24.10	1.052	0.473	
Lower-Middle	893	26.54	1.136	0.468		1283	24.87	1.094	0.451	
Upper-Middle	823	24.46	1.222	0.505		1320	25.59	1.168	0.473	
Highest	873	25.94	1.247	0.501		1312	25.44	1.220	0.481	
Occupation					<.001					<.001
White	1155	34.32	1.229	0.498		1254	24.31	1.160	0.460	
Pink	431	12.81	1.251	0.478		848	16.44	1.155	0.491	
Blue	1186	35.25	1.144	0.489		762	14.77	1.071	0.441	
Others	593	17.62	1.121	0.539		2294	44.47	1.135	0.484	
Alcohol Consumption					0.202					0.222
Yes	3261	96.91	1.185	0.502		4568	88.56	1.138	0.474	
No	104	3.09	1.121	0.513		590	11.44	1.112	0.475	
Cigarettes					0.055					0.557
Smoker	1382	41.07	1.180	0.524		273	5.29	1.123	0.538	
Ex-Smoker	1158	34.41	1.163	0.486		288	5.58	1.109	0.478	
Non-Smoker	825	24.52	1.217	0.486		4597	89.12	1.137	0.470	
Stress Level					0.016					0.247
High	836	24.84	1.219	0.522		1360	26.37	1.120	0.501	
Medium	2048	60.86	1.178	0.501		3170	61.46	1.137	0.460	
Low	481	14.29	1.139	0.468		628	12.18	1.157	0.483	
Nutritional Education					0.741					0.124
No	3247	96.49	1.182	0.501		4947	95.91	1.133	0.474	
Yes	118	3.51	1.198	0.526		211	4.09	1.184	0.480	
Nutrition Supplement Intake (more than2 weeks/year)					<.001					<.001
No	2065	61.37	1.145	0.487		2540	49.24	1.091	0.461	
Yes	1300	38.63	1.242	0.520		2618	50.76	1.178	0.482	
Nutritional Fact Usage		0.00			<.001					<.001
No	2770	82.32	1.157	0.485		3225	62.52	1.102	0.472	
Yes	595	17.68	1.305	0.558		1933	37.48	1.189	0.471	
Total	3365	100.00	1.182	0.501		5158	100.00	1.133	0.471	

The results of regression analyses of the association between eating behaviour and MAR are presented in Table 2. Demographic and socioeconomic information, health status, and nutrition-related variables were adjusted for the analysis. The comparison between ‘some together’ and ‘alone’ showed that the ‘alone’ group’s diet quality was lower than the ‘together’ group as measured by MAR (male: β: − 0.110, *p* = 0.002; female: β: − 0.069, *p* = 0.005). The association between age groups and MAR results are different by gender that male participants have higher MAR than female participants. In male participants, age group and MAR shows positive correlation with statistically significant values while in female participants the negative correlation is detected (male age group 19–29: β: 0.305, *p* < 0.001; male age group 30–39: β: 0.259, *p* < 0.001; male age group 40–49: β: 0.137, *p* < 0.001; male age group 50–59: β: − 0.052, p:0.086; female age group 19–29: β: − 0.035, *p* = 0.346; female age group 30–39: β: − 0.010, *p* = 0.725; male age group 40–49: β: − 0.074, *p* = 0.006; male age group 50–59: β: 0.001 p: 0.981). The living arrangement did not influence the MAR, neither before nor after adjustment. As per stress level, MAR and stress level shows positive correlation among male participants and negative correlation among female participants (male high: β: 0.023, p: 0.423; male medium: β: 0.003, *p* = 0.901; female high: β: − 0.044, *p* = 0.053; female medium: β: − 0.040, *p* = 0.053). People who take nutritional supplement more than 2 weeks per year show higher MAR then who does not take supplements (male: β: − 0.021, *p* = 0.643; female: β: − 0.019, *p* = 0.567) but the value is not statistically significant. Participants who does not use nutritional facts appear to have lower MAR in both gender with significant value (male: β: − 0.087, *p* < 0.001; female: β: − 0.056, *p* < 0.001). (Table 2). In addition to Tables 1 and 2, the general characteristics of the study population according to eating behabiour and the analysis of unadjusted model is presented in Additional file 1: Appendix 1 and 2.

**Table 2:** Factors associated with MAR

Variables	MAR					
	Male			Female		
	β	S.E	*p*-value	β	S.E	*p*-value
Eating Style						
Alone	−0.110	0.035	0.002	− 0.069	0.024	0.005
Some Together	−0.001	0.018	0.936	−0.010	0.014	0.474
Together	Ref.			Ref.		
One person household						
Yes	0.042	0.037	0.262	−0.040	0.032	0.221
No	Ref.			Ref.		
Age Group						
19–29	0.305	0.045	<.001	−0.035	0.037	0.346
30–39	0.259	0.036	<.001	−0.010	0.029	0.725
40–49	0.137	0.033	<.001	−0.074	0.027	0.006
50–59	0.052	0.030	0.086	0.001	0.024	0.981
60 above	Ref.			Ref.		
Residential Area						
Urban	0.001	0.023	0.975	− 0.013	0.019	0.469
Rural	Ref.			Ref.		
BMI						
Underweight	− 0.030	0.025	0.610	−0.011	0.028	0.707
Overweight	0.024	0.018	0.015	0.024	0.016	0.125
Normal	Ref.			Ref.		
Weight Changes						
Decreased	−0.035	0.025	0.161	− 0.048	0.021	0.021
Increased	−0.035	0.022	0.274	0.030	0.015	0.047
Same	Ref.			Ref.		
Marital Status						
Married	0.043	0.032	0.178	0.021	0.027	0.435
Once Married	−0.063	0.051	0.219	−0.023	0.035	0.522
Single	Ref.			Ref.		
Education Level						
Primary	−0.048	0.039	0.223	−0.162	0.028	<.001
Secondary	−0.018	0.036	0.614	−0.077	0.027	0.005
Upper Secondary	0.032	0.021	0.124	−0.019	0.017	0.248
Tertiary	Ref.			Ref.		
Income Level						
Lowest	−0.091	0.026	<.001	−0.113	0.020	<.001
Lower-Middle	−0.103	0.024	<.001	−0.097	0.019	<.001
Upper-Middle	−0.027	0.024	0.249	−0.038	0.018	0.040
Highest	Ref.			Ref.		
Occupation						
White Collar	0.049	0.029	0.095	−0.013	0.018	0.478
Pink Collar	0.091	0.032	0.005	0.032	0.019	0.098
Blue Collar	0.065	0.027	0.018	−0.013	0.021	0.528
Others	Ref.			Ref.		
Alcohol Consumption						
Yes	−0.001	0.049	0.977	0.009	0.021	0.679
No	Ref.			Ref.		
Cigarettes						
Smoker	0.002	0.022	0.931	0.034	0.030	0.258
Once Smoked	0.011	0.023	0.628	−0.019	0.029	0.501
Non-Smoker	Ref.			Ref.		
Stress Level						
High	0.023	0.028	0.423	−0.044	0.023	0.053
Medium	0.003	0.025	0.901	−0.040	0.020	0.053
Low	Ref.			Ref.		
Nutritional Education						
No	−0.021	0.046	0.643	−0.019	0.033	0.567
Yes	Ref.			Ref.		
Nutrition Supplement Intake (more than2 weeks/year)						
No	−0.089	0.018	<.001	−0.072	0.013	<.001
Yes	Ref.			Ref.		
Nutritional Fact Usage						
No	−0.087	0.022	<.001	−0.056	0.014	<.001
Yes	Ref.			Ref.		

Adjusted for living arrangement, residential area, BMI, weight changes, marital status, educational level, income level, occupation, alcohol consumption, smoking, stress level, nutritional education, nutrient supplement intake, and nutrition facts usage

Table 3 shows the result of subgroup analysis based on socioeconomic factors living arrangement, income level, education level, and occupation by eating behaviour. For male participants, when they live with others (β: − 0.134, *p* = 0.001); completed with tertiary education (β: − 0.212, *p* < 0.001); earns the lowest (β: − 0.216, *p* = 0.002) or highest income (β: − 0.167, *p* = 0.040); categorize as ‘others’ (β: − 0.193, *p* = 0.006) for occupational status which means not currently working with the eating alone behaviour shows inadequate diet with lower MAR than those who eat together within the same categories. The results of female study are as followed. Similar to male’s results when they live with others (β: − 0.081, *p* = 0.002); completed primary education (β: − 0.163, *p* = 0.003) or secondary education (β: − 0.146, *p* = 0.040); work in pink-collar industry (β: − 0.125, *p* = 0.035) or blue-collar industry (β: − 0.178, *p* = 0.004) have insufficient nutrient intake with they eat alone (Table 3).

**Table 3:** Eating style and MAR by socioeconomic status

Variables	Eating Style						
	Together	Alone			Some Together		
	β	β	S.E	*p*-value	β	S.E	*p*-value
Male							
One person household							
Yes	Ref.	−0.072	0.105	0.495	0.030	0.090	0.742
No	Ref.	−0.134	0.039	0.001	−0.002	0.018	0.919
Education Level							
Primary	Ref.	−0.141	0.091	0.124	0.022	0.062	0.725
Secondary	Ref.	0.019	0.114	0.867	−0.039	0.499	0.499
Upper Secondary	Ref.	−0.036	0.056	0.520	0.000	0.030	0.999
Tertiary	Ref.	−0.212	0.059	<.001	−0.002	0.027	0.934
Income Level							
Lowest	Ref.	−0.216	0.068	0.002	0.024	0.040	0.539
Lower-Middle	Ref.	−0.045	0.063	0.479	0.010	0.033	0.752
Upper-Middle	Ref.	−0.058	0.079	0.462	−0.008	0.037	0.827
Highest	Ref.	−0.167	0.081	0.040	−0.032	0.036	0.367
Occupation							
White Collar	Ref.	−0.003	0.085	0.970	0.030	0.031	0.343
Pink Collar	Ref.	−0.067	0.085	0.435	−0.123	0.050	0.015
Blue Collar	Ref.	−0.112	0.059	0.057	0.015	0.029	0.595
Others	Ref.	−0.193	0.070	0.006	−0.007	0.046	0.887
Female							
One person household							
Yes	Ref.	0.059	0.092	0.519	0.103	0.086	0.234
No	Ref.	−0.081	0.026	0.002	−0.010	0.014	0.475
Education Level							
Primary	Ref.	−0.163	0.055	0.003	−0.040	0.039	0.309
Secondary	Ref.	−0.146	0.071	0.040	−0.068	0.045	0.128
Upper Secondary	Ref.	−0.048	0.042	0.252	0.009	0.022	0.695
Tertiary	Ref.	−0.024	0.043	0.578	−0.008	0.023	0.710
Income Level							
Lowest	Ref.	−0.077	0.046	0.096	−0.020	0.030	0.501
Lower-Middle	Ref.	−0.083	0.048	0.086	−0.010	0.027	0.702
Upper-Middle	Ref.	−0.086	0.051	0.092	−0.041	0.028	0.135
Highest	Ref.	−0.044	0.052	0.400	0.031	0.028	0.271
Occupation							
White Collar	Ref.	−0.026	0.052	0.622	−0.002	0.027	0.950
Pink Collar	Ref.	−0.125	0.059	0.035	−0.016	0.036	0.669
Blue Collar	Ref.	−0.178	0.062	0.004	−0.055	0.034	0.107
Others	Ref.	−0.038	0.037	0.306	−0.006	0.021	0.795

Adjusted for living arrangement, residential area, BMI, weight changes, marital status, educational level, income level, occupation, alcohol consumption, smoking, stress level, nutritional education, nutrient supplement intake, and nutrition facts usage

**Fig. 1:**
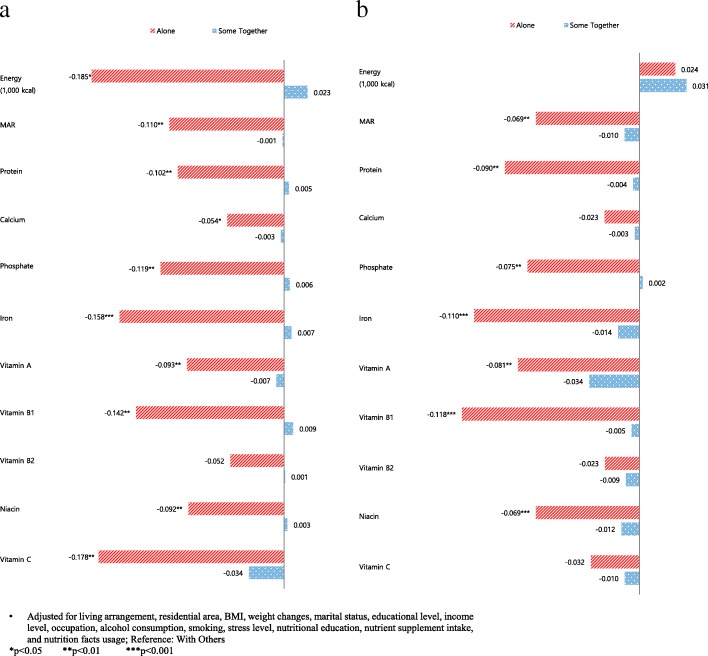
**a** Energy, MAR, and NAR per day in men. **b** Energy, MAR, and NAR per day in women. Alone, Some together, Energy (1000 kcal), MAR Protein, Protein, Calcium, Phosphate, Iron, Vitamin A, Vitamin B1, Vitamin B2, Niacin, Vitamin

Nutrient intake was measured from the dietary survey and was used to assess patterns of food consumption in the previous year. Figure 1 shows the MAR and NAR of individual nutrients by eating behaviour and sex. Regardless of sex, participants consumed inadequate nutrients when they ate alone (Fig. 1). Detailed results on individual 9 nutrients are presented in Additional file 1: Appendix 3. Also, additional analysis such as MAR with the cutoff value of 0.5, different models related to socioeconomic factors, and interactions are included in Additional file 2: Appendix 4, 5 and 6.

## Discussion

The results from this study show that diet quality is influenced by eating behaviour, as indicated by the association between MAR and eating alone. We observed that when Korean adults ate without a companion, their MAR was significantly lower than those who consistently ate with others. Poor quality meals in Korean adults could possibly lead to “modern malnutrition” that not receiving adequate nutrients yet reached the recommended total calories per day. This could result in further health-related problems [5, 10]. When MAR is equal to 1 or above it means that in average the individual consumed the recommended amount of nutrients. However, since it is the average value, further observation of nutrient intake should be performed to decide the quality of the diet. Especially, modern days, there are people who exceed the recommended food intake amount through high-calorie foods and unbalanced diet which lead to poor diet quality.

For socioeconomic element covariates, we chose education level, income level, and occupation variables to identify the vulnerable class to provide social support. Also, those factors could be related to the nutrient intake. After reviewing the interaction between education level, income level, and occupation, we have performed analyses to find the interaction between those variables. As the results, the interactions existed therefore, we used a stratified variable.

After the main analysis, we studied in more depth the association between socioeconomic factors of Korean adults and their diets. We performed subgroup analyses for living arrangement, education level, income level, and occupation status to investigate what other factors could influence diet quality beyond demographic factors [2]. The outcomes of the analyses conflicted with social norms and common beliefs, [19, 23, 25] yet they represent a current social phenomenon in Korea [29]. These results could be applicable as a version of modern society in other countries as well. Our findings indicate that Korean male adults who eat alone have a poor diet with inadequate nutrient intake compared to when they live with others; this finding is consistent in subgroups who have a tertiary education, have the lowest or highest income level, and are part of the ‘others’ group in occupation classification, which includes those who are not currently working. From our study, we also found the result showing gender difference. Men were showing positive correlation to MAR while women were showing negative in age group and stress level. We interpreted the results that it is possible that gender difference could be related to the result. We reviewed previous studies and found that that men and women have different patterns and choices related to food consumption that men choose more toward meat products while women are more in vegetables [30–32]. Like their findings, our study shows vitamins such as Vitamin A, Vitamin B1and Vitamin C intakes were higher in women. Regarding the stress level related to MAR, there are many discussions that men and women have a different stress coping mechanism [33–35]. Also, stress and diet quality has an association that stress can influence eating patterns [36, 37]. Due to the stress, some people changes their eating habits and eat more and some eat less and even develop an eating disorder [38]. Therefore, our results could be led by gender differences in stress coping strategies by eating patterns.

With the result of the subgroup analysis, we discovered that the living arrangement for male and female does not influence the diet quality. As per socioeconomic factors, occupation status shows an association between diet qualities. Compared to men who have an office job, men in the service industry, manual work, and others show a gradual decline in diet quality when they eat alone. In the female population, education level expressed a similar trend. From the higher education level to lower education level, the diet quality declined when they eat alone. In the occupational status, women who are in manual work or service industry showed a significant low diet quality.

MAR which is the mean adequacy ratio is one of the indicators to evaluate the individual’s nutrient intake. To obtain the value of MAR, the nutrient adequacy ratio, NAR is needed. NAR is the measure of a nutrient intake that is corresponding to the recommended dietary allowance (RDA) for the specific gender and age group [27, 28]. Unlike other nutrition indicators, it does not include total individual energy intake. However, it is allowed to express the comprehensiveness of the dietary quality. In measuring individual’s dietary quality, MAR has been considered as a valid indicator as it references to the recommended dietary allowance [39, 40]. Therefore, we used the Korean Recommended Allowance for Nutrients as the reference to calculate NAR. When the NAR or MAR is 1 or above 1, it indicates that the individual has consumed the adequate amount of nutrient that reached RDA. Compared to previous studies, some of our outcomes were similar [20, 23] in men with low income levels. Additionally, related to income, a previous study concluded that people with higher education and income levels will have a better diet because they can afford diet costs [2, 20]. Our results provide another point of view different from the conventional idea of most previous studies; people who have higher socioeconomic status have better meals [2, 20, 41, 42] yet, their diet quality at the individual nutrient level shows insufficient in a certain nutrient.

Our study was conducted with a national survey and the sample is representative of the general Korean population. In addition, previous studies on nutrition were heavily focused on specific generation, while our study population is adults [19, 21, 43]. The volume of research on eating alone has increased as it has become a social issue that is very relevant in today’s society. However, there are hardly any studies evaluating an association between eating behaviour and diet quality through MAR, so this is one of the advantages of this study. The outcomes from our study provide another perspective on the association between socioeconomic status and diet quality. To determine overall diet quality we used MAR, which was calculated by the NAR for each of nine nutrients. This allowed us to view specific details about diet quality as the NAR indicates the exact amount of each nutrient in the diet. In addition, the simple calculation of MAR is composed of micronutrients that provide more detailed nutrient intake and quality information than categorized food groups such as Healthy Eating Index [44].

While there are advantages compared to other studies, there are also several limitations that require investigation through further study. First, there are limitations inherent in a cross-sectional study design and use of survey data. A causal relationship between variables cannot be determined, unlike a study conducted through cohort data [25] in health survey data and nutrition survey data. Therefore, the cause and effect could be vice versa. Also, we might not include all the possible cofounders for the study. In addition, from the nutrition survey, we cannot ensure participants answered with an exact awareness of their food consumption history. The survey was designed to answer for the past 1 year of dietary data rather than 24-h recall data. This would lead to recall bias. Also, the question, by asking for the average of 1 week of the past 1 year to provide supportive evidence to lower the potential bias. This allowed the survey to get general information regarding dietary habits. In this study, carbohydrate and fat did not calculate for NAR and MAR. Based on the methods of KNHANES data on nutrients intake, it was not able to measure NAR for fat and carbohydrates. To calculate NAR, the measurement of units of denominator and numerator should be comparable. In this study, the unit of denominator value is the Recommended Dietary Allowance (RDA) which is reported by The Korean Nutrition Society and The Ministry of Health and Welfare of Korea announce the RDA for the Korean population. In the report, the fat and carbohydrate are given in the Acceptable Macronutrient Distribution Range (AMDR) while the survey, the numerator is collected in the unit of a gram per day. Therefore, matching those two different units of measure to calculate the NAR or MAR was limited when NAR and MAR require to measure the amount of consumed nutrient. Therefore, we reviewed the adequate nutrient intake of nine nutrients and level of energy [26, 29]. Many studies using MAR and NAR as the outcome values, they do not have the exact same nutrients or all macronutrients to evaluate the level of MAR. Previous investigations [22, 45, 46] in the Korean population, they also excluded carbohydrates and fats measures. Those studies included protein, calcium, phosphorus, iron vitamin A, vitamin B, vitamin B1, vitamin B2, niacin, Vitamin C which was the same as our nutrients. In addition, the recent study done by Donna B. Johnson et al. [47] to assess the nutritional quality of adolescents in US Washington State. In that study, they included calcium, vitamin C, vitamin A, iron, fibre, and protein to measure MAR*.* As the MAR is the mean value of adequacy of nutrients, the choice of nutrients is dependent on the researchers. Also, due to the data source, we were able to collect nine nutrients and energy to be studied. Therefore, we suggest conducting comprehensive studies with other nutrients such as carbohydrates, fats, fibre, sodium, and other minerals. We measured diet quality with MAR, which is the average nutrient intake. Since it represents the average amount of adequate nutrient intake ratio, it might be misinterpreted that when the MAR equal to 1. There is a possibility that some of the nutrients are compensating for each other in the MAR. Therefore, the investigation of each nutrient would be helpful. To overcome this type of error, we prepared supplementary tables (Additional file 1: Appendix 3) to see the NAR for each nutrient and noticed certain nutrients are below the recommendations such as calcium, vitamin A, and niacin.

In 2015, the one-person household was reported at 27.2% of the total population of Korea, compared to 15.2% in 2000. It is reasonable to expect that the prevalence of persons eating alone will also increase [24]. The Korea Statistics projects that, in 2045, one-person households will make up 36.6% of the total population. It has also been forecasted that the elderly population will expand as well [14, 24]. Based on these numbers, it is not hard to expect that solitary eating will become a common way of eating. Many studies warn that a poor diet could lead to serious health problems [10–12] and emphasize the importance of quality meals [9, 23]. People today experience “modern malnutrition”, which is caused by modern diet habits such as high intakes of sugar, fat, fast food, and soda [8, 11, 48–50]. This type of diet is typically found in solitary eating. If people continue to eat alone, they will have potential risks of developing obesity and metabolic syndromes [11, 45].

The new social phenomena of an increase in one-person households creates public isolation, which can affect public health [14], but at the same time, we need to acknowledge that we have been ignoring the potential risk that another party could have. We were able to infer that, along with changes in our daily lifestyle, products that supplement diet balance for people who live alone may be beneficial. However, people who live together, mostly with family [25] consider their meals better than others even when they eat alone. Similar to that idea, people who are in a higher social stratum are considered more able to maintain their health by themselves. As we discovered from our analyses, poor meal quality does not appear only among the socially disadvantaged or those who live alone. The results indicate that we should promote nutritional health awareness to the lower socioeconomic class yet we should discriminate to improve diet quality for the entire population as the result of subgroup analysis shown.

## Conclusions

This study provides evidence to promote interventions to improve the quality of the diet of the public. Many Korean adults are experiencing low diet quality when they eat alone. The number of people who eat alone is increasing along with the changes of lifestyle. Also, the people who were considered as upper socio-economic status, such as who have high income, education level and white collar occupation status, are also experiencing issues in diet quality. Our study is highly recommended to policy-makers to utilize it as evidence to develop and improve social welfare services for the general population.

## Additional file

Additional file 1: **Appendix 1.** Characteristics of study population by eating behaviour **Appendix 2.** Results of unadjusted and adjusted multiple regression associated with MAR **Appendix 3.** NAR of nutrients and total energy intake. (DOCX 140 kb)

Additional file 2: **Appendix 4.** Association between variables and MAR by cutoff value at 0.5 **Appendix 5.** Results of adjusted and unadjusted model related to socioeconomic factors **Appendix 6.** Interaction between variables. (DOCX 24.3 kb)

## Abbreviations

BMI: Body mass index
GLM: Generalized linear model
KNHANES: Korean National Health and Nutrition Examination Survey
MAR: Mean adequacy ratio
NAR: The nutrient adequacy ratio
RDA: Recommended dietary allowance
