# Supplementary material for: Association between eating behaviour and diet quality: eating alone vs. eating with others
Source: Nutr J. 2018 Dec 19;17:117. doi: 10.1186/s12937-018-0424-0 (PMC6299993; doi:10.1186/s12937-018-0424-0)
Supplement: Supplementary file 1 — Appendix 1. Characteristics of study population by eating behaviour Appendix 2. Results of unadjusted and adjusted multiple regression associated with MAR Appendix 3. NAR of nutrients and total energy intake. (DOCX 140 kb) [file 12937_2018_424_MOESM1_ESM.docx]

| Appendix 1. Characteristics of study population by eating behaviour | | | | | | |
| --- | --- | --- | --- | --- | --- | --- |
| **Variables** | **Eating Alone** | | **Some Together** | | **Together** | |
|  | **N** | **%** | **N** | **%** | **N** | **%** |
| **Living Arrangement** |  |  |  |  |  |  |
| Living Alone | 211 | 27.8 | 209 | 6.1 | 108 | 2.5 |
| Living with Others | 547 | 72.2 | 3245 | 94.0 | 4203 | 97.5 |
| **Age Group** |  |  |  |  |  |  |
| 19 - 29 | 137 | 18.1 | 531 | 15.4 | 710 | 16.5 |
| 30 - 39 | 92 | 12.1 | 722 | 20.9 | 1021 | 23.7 |
| 40 - 49 | 126 | 16.6 | 887 | 25.7 | 1076 | 25.0 |
| 50 - 59 | 257 | 33.9 | 923 | 26.7 | 1022 | 23.7 |
| 60 above | 146 | 19.3 | 391 | 11.3 | 482 | 11.2 |
| **Residential Area** |  |  |  |  |  |  |
| Urban | 663 | 87.5 | 2985 | 86.4 | 3487 | 80.9 |
| Rural | 95 | 12.5 | 469 | 13.6 | 824 | 19.1 |
| **BMI** |  |  |  |  |  |  |
| Underweight | 36 | 4.8 | 162 | 4.7 | 187 | 4.3 |
| Normal | 469 | 61.9 | 2260 | 65.4 | 2733 | 63.4 |
| Overweight | 253 | 33.4 | 1032 | 29.9 | 1391 | 32.3 |
| **Weight Changes** |  |  |  |  |  |  |
| Same | 429 | 56.6 | 2091 | 60.5 | 2643 | 61.3 |
| Decreased | 117 | 15.4 | 429 | 12.4 | 534 | 12.4 |
| Increased | 212 | 28.0 | 934 | 27.0 | 1134 | 26.3 |
| **Marital Status** |  |  |  |  |  |  |
| Married | 343 | 45.3 | 2538 | 73.5 | 3297 | 76.5 |
| Separated | 212 | 28.0 | 255 | 7.4 | 181 | 4.2 |
| Single | 203 | 26.8 | 661 | 19.1 | 833 | 19.3 |
| **Education Level** |  |  |  |  |  |  |
| Primary | 169 | 22.3 | 339 | 9.8 | 393 | 9.1 |
| Secondary | 96 | 12.7 | 325 | 9.4 | 384 | 8.9 |
| Upper Secondary | 263 | 34.7 | 1395 | 40.4 | 1697 | 39.4 |
| Tertiary | 230 | 30.3 | 1395 | 40.4 | 1837 | 42.6 |
| **Income Level** |  |  |  |  |  |  |
| Lowest | 284 | 37.5 | 755 | 21.9 | 980 | 22.7 |
| Lower-Middle | 182 | 24.0 | 896 | 25.9 | 1098 | 25.5 |
| Upper-Middle | 148 | 19.5 | 907 | 26.3 | 1088 | 25.2 |
| Highest | 144 | 19.0 | 896 | 25.9 | 1145 | 26.6 |
| **Occupation** |  |  |  |  |  |  |
| White | 124 | 16.4 | 864 | 25.0 | 1421 | 33.0 |
| Pink | 131 | 17.3 | 462 | 13.4 | 686 | 15.9 |
| Blue | 153 | 20.2 | 753 | 21.8 | 1042 | 24.2 |
| Others | 350 | 46.2 | 1375 | 39.8 | 1162 | 27.0 |
| **Alcohol Consumption** |  |  |  |  |  |  |
| Yes | 685 | 90.4 | 3164 | 91.6 | 3980 | 92.3 |
| No | 73 | 9.6 | 290 | 8.4 | 331 | 7.7 |
| **Cigarettes** |  |  |  |  |  |  |
| Smoker | 165 | 21.8 | 574 | 16.6 | 916 | 21.3 |
| Ex-Smoker | 115 | 15.2 | 530 | 15.3 | 801 | 18.6 |
| Non-Smoker | 478 | 63.1 | 2350 | 68.0 | 2594 | 60.2 |
| **Stress Level** |  |  |  |  |  |  |
| High | 240 | 31.7 | 841 | 24.4 | 1115 | 25.9 |
| Medium | 406 | 53.6 | 2135 | 61.8 | 2677 | 62.1 |
| Low | 112 | 14.8 | 478 | 13.8 | 519 | 12.0 |
| **Nutritional Education** |  |  |  |  |  |  |
| No | 731 | 96.4 | 3319 | 96.1 | 4144 | 96.1 |
| Yes | 73 | 9.6 | 135 | 3.9 | 167 | 3.9 |
| **Nutrition Supplement Intake (more than2 weeks/year)** |  |  |  |  |  |  |
| No | 354 | 46.7 | 1629 | 47.2 | 1935 | 44.9 |
| Yes | 404 | 53.3 | 1825 | 52.8 | 2376 | 55.1 |
| **Nutritional Fact Usage** |  |  |  |  |  |  |
| No | 210 | 27.7 | 1093 | 31.6 | 1225 | 28.4 |
| Yes | 548 | 72.3 | 2361 | 68.4 | 3086 | 71.6 |
| **Total** | 758 | 100.0 | 3454 | 100.0 | 4311 | 100.0 |

| Appendix 2. Results of unadjusted and adjusted multiple regression associated with MAR | | | | | | | |
| --- | --- | --- | --- | --- | --- | --- | --- |
|  | **Eating Style** | | | | | | |
|  | **Alone** | | | **Some Together** | | | **Together** |
|  | **β** | **S.E** | ***p*-VALUE** | **β** | **S.E** | ***p*-VALUE** | **β** |
| Model 1 | -0.137 | 0.019 | <.001 | -0.013 | 0.011 | 0.226 | Ref. |
| Model 2 | -0.114 | 0.019 | <.001 | -0.005 | 0.011 | 0.632 | Ref. |
| Model 3 | -0.113 | 0.019 | <.001 | -0.008 | 0.011 | 0.485 | Ref. |
| Model 4 | -0.079 | 0.020 | <.001 | -0.003 | 0.011 | 0.759 | Ref. |
| Model 1: Unadjusted | | | | | | | |
| Model 2: Adjusted for age and sex | | | | | | | |
| Model 3: Adjusted for sex, age, and health (behavior) factors | | | | | | | |
| Model 4: Adjusted for sex, age, and socio-economic factors | | | | | | | |

| Appendix 3. NAR of nutrients and total energy intake | | | | | | | |  | |  | | |  | |  |  | |  | | |  | |  |  | | | | | | |
| --- | --- | --- | --- | --- | --- | --- | --- | --- | --- | --- | --- | --- | --- | --- | --- | --- | --- | --- | --- | --- | --- | --- | --- | --- | --- | --- | --- | --- | --- | --- |
| **Variables** | |  | | |  |  | |  | | **Baseline characteristics** | | | | | | | | | | | | | | | | | | | | |
|  |  | **Energy (kcal)** | | | | | | | | **NAR Protein** | | | | | | | | **NAR Calcium** | | | | | | | | | | | | |
|  |  | **MEAN** | | | **±** | **SD** | | ***p*-VALUE** | | **MEAN** | | | **±** | | **SD** | ***p*-VALUE** | | **MEAN** | | | **±** | | **SD** | ***p*-VALUE** | | | | | | |
| **Eating Style** | |  | | |  |  | | 0.016 | |  | | |  | |  | <.001 | |  | | |  | |  | <.001 | | | | | | |
| Alone | | 2069.15 | | | ± | 915.93 | |  | | 1.12 | | | ± | | 0.49 |  | | 0.60 | | | ± | | 0.32 |  | | | | | | |
| Some together | | 2147.65 | | | ± | 958.64 | |  | | 1.25 | | | ± | | 0.54 |  | | 0.66 | | | ± | | 0.31 |  | | | | | | |
| Together | | 2185.55 | | | ± | 939.26 | |  | | 1.25 | | | ± | | 0.52 |  | | 0.65 | | | ± | | 0.29 |  | | | | | | |
| **Living Arrangement** | |  | | |  |  | | 0.284 | |  | | |  | |  | 0.009 | |  | | |  | |  | 0.047 | | | | | | |
| Living Alone | | 2238.24 | | | ± | 1084.43 | |  | | 1.16 | | | ± | | 0.57 |  | | 0.62 | | | ± | | 0.33 |  | | | | | | |
| Living with Others | | 2166.92 | | | ± | 957.28 | |  | | 1.25 | | | ± | | 0.53 |  | | 0.66 | | | ± | | 0.30 |  | | | | | | |
| **Gender** | |  | | |  |  | | <.001 | |  | | |  | |  | 0.312 | |  | | |  | |  |  | | | | | | |
| Male | | 2597.40 | | | ± | 1096.14 | |  | | 1.26 | | | ± | | 0.55 |  | | 0.67 | | | ± | | 0.31 |  | | | | | | |
| Female | | 1865.84 | | | ± | 714.60 | |  | | 1.24 | | | ± | | 0.51 |  | | 0.64 | | | ± | | 0.30 |  | | | | | | |
| **Age Group** | |  | | |  |  | | <.001 | |  | | |  | |  | <.001 | |  | | |  | |  | 0.002 | | | | | | |
| 19 – 29 | | 2389.69 | | | ± | 1099.14 | |  | | 1.45 | | | ± | | 0.60 |  | | 0.77 | | | ± | | 0.34 |  | | | | | | |
| 30 – 39 | | 2279.76 | | | ± | 1033.02 | |  | | 1.48 | | | ± | | 0.60 |  | | 0.75 | | | ± | | 0.33 |  | | | | | | |
| 40 – 49 | | 2193.69 | | | ± | 951.68 | |  | | 1.29 | | | ± | | 0.52 |  | | 0.68 | | | ± | | 0.30 |  | | | | | | |
| 50 – 59 | | 2109.37 | | | ± | 964.44 | |  | | 1.13 | | | ± | | 0.45 |  | | 0.60 | | | ± | | 0.27 |  | | | | | | |
| 60 above | | 1990.22 | | | ± | 746.21 | |  | | 1.01 | | | ± | | 0.38 |  | | 0.56 | | | ± | | 0.26 |  | | | | | | |
| **Residential Area** | |  | | |  |  | | 0.101 | |  | | |  | |  | <.001 | |  | | |  | |  | <.001 | | | | | | |
| Urban | | 2159.14 | | | ± | 946.23 | |  | | 1.26 | | | ± | | 0.53 |  | | 0.66 | | | ± | | 0.31 |  | | | | | | |
| Rural | | 2215.99 | | | ± | 1030.85 | |  | | 1.19 | | | ± | | 0.50 |  | | 0.62 | | | ± | | 0.30 |  | | | | | | |
| **BMI** | |  | | |  |  | | 0.002 | |  | | |  | |  | 0.762 | |  | | |  | |  | <.001 | | | | | | |
| Underweight | | 1960.62 | | | ± | 713.87 | |  | | 1.26 | | | ± | | 0.54 |  | | 0.64 | | | ± | | 0.33 |  | | | | | | |
| Normal | | 2158.78 | | | ± | 951.67 | |  | | 1.24 | | | ± | | 0.51 |  | | 0.66 | | | ± | | 0.30 |  | | | | | | |
| Overweight | | 2216.96 | | | ± | 1007.02 | |  | | 1.25 | | | ± | | 0.56 |  | | 0.66 | | | ± | | 0.31 |  | | | | | | |
| **Weight Changes** | |  | | |  |  | | 0.014 | |  | | |  | |  | <.001 | |  | | |  | |  | 0.8277 | | | | | | |
| Same | | 2195.58 | | | ± | 959.78 | |  | | 1.23 | | | ± | | 0.50 |  | | 0.65 | | | ± | | 0.29 |  | | | | | | |
| Decreased | | 2165.54 | | | ± | 966.63 | |  | | 1.18 | | | ± | | 0.53 |  | | 0.63 | | | ± | | 0.31 |  | | | | | | |
| Increased | | 2097.75 | | | ± | 968.66 | |  | | 1.34 | | | ± | | 0.59 |  | | 0.70 | | | ± | | 0.33 |  | | | | | | |
| **Marital Status** | |  | | |  |  | | <.001 | |  | | |  | |  | <.001 | |  | | |  | |  | <.001 | | | | | | |
| Married | | 2149.99 | | | ± | 940.01 | |  | | 1.23 | | | ± | | 0.51 |  | | 0.65 | | | ± | | 0.30 |  | | | | | | |
| Once Married | | 1978.48 | | | ± | 832.84 | |  | | 1.09 | | | ± | | 0.43 |  | | 0.55 | | | ± | | 0.25 |  | | | | | | |
| Single | | 2370.50 | | | ± | 1107.54 | |  | | 1.43 | | | ± | | 0.64 |  | | 0.75 | | | ± | | 0.34 |  | | | | | | |
| **Education Level** | |  | | |  |  | | <.001 | |  | | |  | |  | <.001 | |  | | |  | |  | <.001 | | | | | | |
| Primary | | 1866.09 | | | ± | 774.17 | |  | | 0.98 | | | ± | | 0.39 |  | | 0.52 | | | ± | | 0.25 |  | | | | | | |
| Secondary | | 1993.42 | | | ± | 797.98 | |  | | 1.10 | | | ± | | 0.44 |  | | 0.59 | | | ± | | 0.28 |  | | | | | | |
| Upper Secondary | | 2228.34 | | | ± | 1064.56 | |  | | 1.29 | | | ± | | 0.55 |  | | 0.68 | | | ± | | 0.32 |  | | | | | | |
| Tertiary | | 2266.99 | | | ± | 930.72 | |  | | 1.33 | | | ± | | 0.54 |  | | 0.70 | | | ± | | 0.30 |  | | | | | | |
| **Income Level** | |  | | |  |  | | <.001 | |  | | |  | |  | <.001 | |  | | |  | |  | <.001 | | | | | | |
| Lowest | | 2090.26 | | | ± | 936.53 | |  | | 1.19 | | | ± | | 0.54 |  | | 0.62 | | | ± | | 0.30 |  | | | | | | |
| Lower-Middle | | 2163.15 | | | ± | 926.41 | |  | | 1.21 | | | ± | | 0.51 |  | | 0.64 | | | ± | | 0.30 |  | | | | | | |
| Upper-Middle | | 2137.43 | | | ± | 873.53 | |  | | 1.28 | | | ± | | 0.53 |  | | 0.66 | | | ± | | 0.30 |  | | | | | | |
| Highest | | 2273.55 | | | ± | 1083.17 | |  | | 1.30 | | | ± | | 0.53 |  | | 0.70 | | | ± | | 0.30 |  | | | | | | |
| **Occupation** | |  | | |  |  | | <.001 | |  | | |  | |  | <.001 | |  | | |  | |  | <.001 | | | | | | |
| White | | 2317.91 | | | ± | 951.88 | |  | | 1.32 | | | ± | | 0.52 |  | | 0.69 | | | ± | | 0.30 |  | | | | | | |
| Pink | | 2116.71 | | | ± | 890.69 | |  | | 1.28 | | | ± | | 0.52 |  | | 0.67 | | | ± | | 0.29 |  | | | | | | |
| Blue | | 2310.97 | | | ± | 1091.20 | |  | | 1.18 | | | ± | | 0.51 |  | | 0.63 | | | ± | | 0.31 |  | | | | | | |
| Others | | 1958.57 | | | ± | 840.91 | |  | | 1.23 | | | ± | | 0.55 |  | | 0.65 | | | ± | | 0.31 |  | | | | | | |
| **Alcohol Consumption** | |  | | |  |  | | <.001 | |  | | |  | |  | <.001 | |  | | |  | |  | <.001 | | | | | | |
| Yes | | 2199.57 | | | ± | 962.66 | |  | | 1.26 | | | ± | | 0.53 |  | | 0.66 | | | ± | | 0.31 |  | | | | | | |
| No | | 1895.49 | | | ± | 925.35 | |  | | 1.13 | | | ± | | 0.48 |  | | 0.59 | | | ± | | 0.29 |  | | | | | | |
| **Cigarettes** | |  | | |  |  | | <.001 | |  | | |  | |  | 0.036 | |  | | |  | |  | 0.196 | | | | | | |
| Smoker | | 2637.05 | | | ± | 1075.54 | |  | | 1.29 | | | ± | | 0.58 |  | | 0.67 | | | ± | | 0.31 |  | | | | | | |
| Once Smoked | | 2473.99 | | | ± | 1107.99 | |  | | 1.23 | | | ± | | 0.53 |  | | 0.66 | | | ± | | 0.30 |  | | | | | | |
| Non-Smoker | | 1972.79 | | | ± | 819.71 | |  | | 1.24 | | | ± | | 0.52 |  | | 0.65 | | | ± | | 0.30 |  | | | | | | |
| **Stress Level** | |  | | |  |  | | 0.035 | |  | | |  | |  | 0.002 | |  | | |  | |  | 0.221 | | | | | | |
| High | | 2236.86 | | | ± | 1065.25 | |  | | 1.29 | | | ± | | 0.56 |  | | 0.67 | | | ± | | 0.34 |  | | | | | | |
| Medium | | 2152.14 | | | ± | 936.08 | |  | | 1.25 | | | ± | | 0.52 |  | | 0.65 | | | ± | | 0.30 |  | | | | | | |
| Low | | 2148.39 | | | ± | 915.67 | |  | | 1.20 | | | ± | | 0.50 |  | | 0.64 | | | ± | | 0.28 |  | | | | | | |
| **Nutritional Education** | |  | | |  |  | | 0.010 | |  | | |  | |  | 0.586 | |  | | |  | |  | 0.978 | | | | | | |
| Yes | | 2001.46 | | | ± | 813.75 | |  | | 1.23 | | | ± | | 0.54 |  | | 0.66 | | | ± | | 0.29 |  | | | | | | |
| No | | 2177.36 | | | ± | 968.58 | |  | | 1.25 | | | ± | | 0.53 |  | | 0.66 | | | ± | | 0.31 |  | | | | | | |
| **Nutrition Supplement Intake (more than2 weeks/year)** | |  | | |  |  | | 0.414 | |  | | |  | |  | 0.002 | |  | | |  | |  | <.001 | | | | | | |
| Yes | | 2158.39 | | | ± | 888.04 | |  | | 1.27 | | | ± | | 0.53 |  | | 0.68 | | | ± | | 0.31 |  | | | | | | |
| No | | 2180.73 | | | ± | 1027.14 | |  | | 1.22 | | | ± | | 0.53 |  | | 0.63 | | | ± | | 0.30 |  | | | | | | |
| **Nutritional Fact Usage** | |  | | |  |  | | <.001 | |  | | |  | |  | <.001 | |  | | |  | |  | <.001 | | | | | | |
| Yes | | 2046.76 | | | ± | 868.26 | |  | | 1.33 | | | ± | | 0.53 |  | | 0.71 | | | ± | | 0.32 |  | | | | | | |
| No | | 2217.97 | | | ± | 993.68 | |  | | 1.21 | | | ± | | 0.53 |  | | 0.63 | | | ± | | 0.30 |  | | | | | | |
| **Total** | | 2170.07 | | | ± | 963.23 | |  | | 1.25 | | | ± | | 0.53 |  | | 0.66 | | | ± | | 0.30 |  | | | | | | |
|  | |  | | |  |  | |  | |  | | |  | |  |  | |  | | |  | |  |  | | | | | | |
|  | |  | | |  |  | |  | |  | | |  | |  |  | |  | | |  | |  |  | | | | | | |
| Appendix 3. NAR of nutrients and total energy intake continue | | | | | | | | | | | | | | | | | | | | | | | | | | | |  |  |  |
| **Variables** | **Baseline characteristics** | | | | | | | | | | | | | | | | | | | | | | | |  |  |  | | |  |
|  | **NAR Phosphate** | | | | | | | | **NAR Iron** | | | | | | | | **NAR Vitamin A** | | | | | | | |  |  |  | | |  |
|  | **MEAN** | | **±** | **SD** | | | ***p*-VALUE** | | **MEAN** | | **±** | **SD** | | ***p*-VALUE** | | | **MEAN** | | **±** | **SD** | | ***p*-VALUE** | | |  |  |  | |  |  |
| **Eating Style** |  | |  |  | | | <.001 | |  | |  |  | | 0.028 | | |  | |  |  | | 0.001 | | |  |  |  | |  |  |
| Alone | 1.40 | | ± | 0.58 | | |  | | 1.41 | | ± | 0.57 | |  | | | 0.87 | | ± | 0.49 | |  | | |  |  |  | |  |  |
| Some together | 1.51 | | ± | 0.60 | | |  | | 1.42 | | ± | 0.64 | |  | | | 0.97 | | ± | 0.51 | |  | | |  |  |  | |  |  |
| Together | 1.52 | | ± | 0.59 | | |  | | 1.47 | | ± | 0.64 | |  | | | 0.98 | | ± | 0.51 | |  | | |  |  |  | |  |  |
| **Living Arrangement** |  | |  |  | | | 0.084 | |  | |  |  | | 0.066 | | |  | |  |  | | 0.015 | | |  |  |  | |  |  |
| Living Alone | 1.45 | | ± | 0.68 | | |  | | 1.53 | | ± | 0.70 | |  | | | 0.89 | | ± | 0.55 | |  | | |  |  |  | |  |  |
| Living with Others | 1.52 | | ± | 0.59 | | |  | | 1.45 | | ± | 0.64 | |  | | | 0.98 | | ± | 0.51 | |  | | |  |  |  | |  |  |
| **Gender** |  | |  |  | | | <.001 | |  | |  |  | | <.001 | | |  | |  |  | | <.001 | | |  |  |  | |  |  |
| Male | 1.64 | | ± | 0.65 | | |  | | 1.59 | | ± | 0.63 | |  | | | 0.88 | | ± | 0.45 | |  | | |  |  |  | |  |  |
| Female | 1.43 | | ± | 0.53 | | |  | | 1.35 | | ± | 0.63 | |  | | | 1.04 | | ± | 0.54 | |  | | |  |  |  | |  |  |
| **Age Group** |  | |  |  | | | <.001 | |  | |  |  | | <.001 | | |  | |  |  | | <.001 | | |  |  |  | |  |  |
| 19 – 29 | 1.77 | | ± | 0.70 | | |  | | 1.36 | | ± | 0.67 | |  | | | 0.98 | | ± | 0.48 | |  | | |  |  |  | |  |  |
| 30 – 39 | 1.70 | | ± | 0.65 | | |  | | 1.33 | | ± | 0.61 | |  | | | 1.05 | | ± | 0.52 | |  | | |  |  |  | |  |  |
| 40 – 49 | 1.54 | | ± | 0.59 | | |  | | 1.27 | | ± | 0.59 | |  | | | 0.98 | | ± | 0.50 | |  | | |  |  |  | |  |  |
| 50 - 59 | 1.41 | | ± | 0.52 | | |  | | 1.64 | | ± | 0.63 | |  | | | 0.96 | | ± | 0.51 | |  | | |  |  |  | |  |  |
| 60 above | 1.32 | | ± | 0.47 | | |  | | 1.56 | | ± | 0.62 | |  | | | 0.91 | | ± | 0.53 | |  | | |  |  |  | |  |  |
| **Residential Area** |  | |  |  | | | 0.006 | |  | |  |  | | 0.121 | | |  | |  |  | | 0.001 | | |  |  |  | |  |  |
| Urban | 1.53 | | ± | 0.60 | | |  | | 1.44 | | ± | 0.64 | |  | | | 0.98 | | ± | 0.51 | |  | | |  |  |  | |  |  |
| Rural | 1.47 | | ± | 0.57 | | |  | | 1.48 | | ± | 0.64 | |  | | | 0.92 | | ± | 0.49 | |  | | |  |  |  | |  |  |
| **BMI** |  | |  |  | | | 0.006 | |  | |  |  | | <.001 | | |  | |  |  | | 0.306 | | |  |  |  | |  |  |
| Underweight | 1.45 | | ± | 0.57 | | |  | | 1.16 | | ± | 0.56 | |  | | | 0.93 | | ± | 0.51 | |  | | |  |  |  | |  |  |
| Normal | 1.50 | | ± | 0.57 | | |  | | 1.41 | | ± | 0.62 | |  | | | 0.97 | | ± | 0.49 | |  | | |  |  |  | |  |  |
| Overweight | 1.55 | | ± | 0.63 | | |  | | 1.56 | | ± | 0.67 | |  | | | 0.99 | | ± | 0.54 | |  | | |  |  |  | |  |  |
| **Weight Changes** |  | |  |  | | | <.001 | |  | |  |  | | 0.010 | | |  | |  |  | | <.001 | | |  |  |  | |  |  |
| Same | 1.50 | | ± | 0.57 | | |  | | 1.46 | | ± | 0.63 | |  | | | 0.96 | | ± | 0.50 | |  | | |  |  |  | |  |  |
| Decreased | 1.46 | | ± | 0.61 | | |  | | 1.38 | | ± | 0.62 | |  | | | 0.91 | | ± | 0.50 | |  | | |  |  |  | |  |  |
| Increased | 1.59 | | ± | 0.64 | | |  | | 1.44 | | ± | 0.67 | |  | | | 1.03 | | ± | 0.55 | |  | | |  |  |  | |  |  |
| **Marital Status** |  | |  |  | | | <.001 | |  | |  |  | | 0.016 | | |  | |  |  | | <.001 | | |  |  |  | |  |  |
| Married | 1.49 | | ± | 0.57 | | |  | | 1.46 | | ± | 0.64 | |  | | | 0.98 | | ± | 0.52 | |  | | |  |  |  | |  |  |
| Once Married | 1.31 | | ± | 0.47 | | |  | | 1.43 | | ± | 0.58 | |  | | | 0.85 | | ± | 0.44 | |  | | |  |  |  | |  |  |
| Single | 1.74 | | ± | 0.72 | | |  | | 1.39 | | ± | 0.68 | |  | | | 0.98 | | ± | 0.50 | |  | | |  |  |  | |  |  |
| **Education Level** |  | |  |  | | | <.001 | |  | |  |  | | <.001 | | |  | |  |  | | <.001 | | |  |  |  | |  |  |
| Primary | 1.24 | | ± | 0.48 | | |  | | 1.50 | | ± | 0.62 | |  | | | 0.84 | | ± | 0.48 | |  | | |  |  |  | |  |  |
| Secondary | 1.38 | | ± | 0.50 | | |  | | 1.56 | | ± | 0.63 | |  | | | 0.93 | | ± | 0.51 | |  | | |  |  |  | |  |  |
| Upper Secondary | 1.57 | | ± | 0.62 | | |  | | 1.46 | | ± | 0.66 | |  | | | 0.99 | | ± | 0.51 | |  | | |  |  |  | |  |  |
| Tertiary | 1.60 | | ± | 0.59 | | |  | | 1.39 | | ± | 0.63 | |  | | | 1.01 | | ± | 0.52 | |  | | |  |  |  | |  |  |
| **Income Level** |  | |  |  | | | <.001 | |  | |  |  | | <.001 | | |  | |  |  | | <.001 | | |  |  |  | |  |  |
| Lowest | 1.45 | | ± | 0.62 | | |  | | 1.40 | | ± | 0.66 | |  | | | 0.91 | | ± | 0.51 | |  | | |  |  |  | |  |  |
| Lower-Middle | 1.49 | | ± | 0.58 | | |  | | 1.41 | | ± | 0.60 | |  | | | 0.92 | | ± | 0.48 | |  | | |  |  |  | |  |  |
| Upper-Middle | 1.54 | | ± | 0.59 | | |  | | 1.46 | | ± | 0.62 | |  | | | 0.99 | | ± | 0.49 | |  | | |  |  |  | |  |  |
| Highest | 1.58 | | ± | 0.59 | | |  | | 1.52 | | ± | 0.66 | |  | | | 1.05 | | ± | 0.55 | |  | | |  |  |  | |  |  |
| **Occupation** |  | |  |  | | | <.001 | |  | |  |  | | <.001 | | |  | |  |  | | <.001 | | |  |  |  | |  |  |
| White | 1.60 | | ± | 0.58 | | |  | | 1.42 | | ± | 0.62 | |  | | | 0.99 | | ± | 0.49 | |  | | |  |  |  | |  |  |
| Pink | 1.53 | | ± | 0.58 | | |  | | 1.52 | | ± | 0.65 | |  | | | 1.01 | | ± | 0.50 | |  | | |  |  |  | |  |  |
| Blue | 1.50 | | ± | 0.61 | | |  | | 1.53 | | ± | 0.64 | |  | | | 0.89 | | ± | 0.47 | |  | | |  |  |  | |  |  |
| Others | 1.46 | | ± | 0.59 | | |  | | 1.39 | | ± | 0.64 | |  | | | 1.00 | | ± | 0.56 | |  | | |  |  |  | |  |  |
| **Alcohol Consumption** |  | |  |  | | | <.001 | |  | |  |  | | 0.012 | | |  | |  |  | | 0.118 | | |  |  |  | |  |  |
| Yes | 1.53 | | ± | 0.60 | | |  | | 1.44 | | ± | 0.63 | |  | | | 0.97 | | ± | 0.50 | |  | | |  |  |  | |  |  |
| No | 1.37 | | ± | 0.55 | | |  | | 1.52 | | ± | 0.70 | |  | | | 1.01 | | ± | 0.57 | |  | | |  |  |  | |  |  |
| **Cigarettes** |  | |  |  | | | <.001 | |  | |  |  | | <.001 | | |  | |  |  | | <.001 | | |  |  |  | |  |  |
| Smoker | 1.65 | | ± | 0.67 | | |  | | 1.56 | | ± | 0.64 | |  | | | 0.89 | | ± | 0.48 | |  | | |  |  |  | |  |  |
| Once Smoked | 1.59 | | ± | 0.62 | | |  | | 1.52 | | ± | 0.61 | |  | | | 0.88 | | ± | 0.44 | |  | | |  |  |  | |  |  |
| Non-Smoker | 1.46 | | ± | 0.56 | | |  | | 1.40 | | ± | 0.64 | |  | | | 1.02 | | ± | 0.53 | |  | | |  |  |  | |  |  |
| **Stress Level** |  | |  |  | | | 0.101 | |  | |  |  | | 0.152 | | |  | |  |  | | 0.5658 | | |  |  |  | |  |  |
| High | 1.55 | | ± | 0.64 | | |  | | 1.44 | | ± | 0.66 | |  | | | 0.98 | | ± | 0.53 | |  | | |  |  |  | |  |  |
| Medium | 1.51 | | ± | 0.58 | | |  | | 1.44 | | ± | 0.63 | |  | | | 0.97 | | ± | 0.51 | |  | | |  |  |  | |  |  |
| Low | 1.49 | | ± | 0.57 | | |  | | 1.49 | | ± | 0.63 | |  | | | 0.96 | | ± | 0.50 | |  | | |  |  |  | |  |  |
| **Nutritional Education** |  | |  |  | | | 0.573 | |  | |  |  | | 0.634 | | |  | |  |  | | 0.905 | | |  |  |  | |  |  |
| Yes | 1.49 | | ± | 0.59 | | |  | | 1.47 | | ± | 0.68 | |  | | | 0.98 | | ± | 0.48 | |  | | |  |  |  | |  |  |
| No | 1.52 | | ± | 0.59 | | |  | | 1.45 | | ± | 0.64 | |  | | | 0.97 | | ± | 0.51 | |  | | |  |  |  | |  |  |
| **Nutrition Supplement Intake (more than2 weeks/year)** |  | |  |  | | | 0.002 | |  | |  |  | | <.001 | | |  | |  |  | | <.001 | | |  |  |  | |  |  |
| Yes | 1.54 | | ± | 0.60 | | |  | | 1.50 | | ± | 0.66 | |  | | | 1.02 | | ± | 0.52 | |  | | |  |  |  | |  |  |
| No | 1.49 | | ± | 0.59 | | |  | | 1.40 | | ± | 0.62 | |  | | | 0.93 | | ± | 0.50 | |  | | |  |  |  | |  |  |
| **Nutritional Fact Usage** |  | |  |  | | | <.001 | |  | |  |  | | <.001 | | |  | |  |  | | <.001 | | |  |  |  | |  |  |
| Yes | 1.58 | | ± | 0.60 | | |  | | 1.40 | | ± | 0.67 | |  | | | 1.08 | | ± | 0.54 | |  | | |  |  |  | |  |  |
| No | 1.49 | | ± | 0.59 | | |  | | 1.47 | | ± | 0.63 | |  | | | 0.93 | | ± | 0.49 | |  | | |  |  |  | |  |  |
| **Total** | 1.52 | | ± | 0.59 | | |  | | 1.45 | | ± | 0.64 | |  | | | 0.97 | | ± | 0.51 | |  | | |  |  |  | |  |  |
|  |  | |  |  | | |  | |  | |  |  | |  | | |  | |  |  | |  | | |  |  |  | |  |  |
|  |  | |  |  | | |  | |  | |  |  | |  | | |  | |  |  | |  | | |  |  |  | |  |  |
|  |  | |  |  | | |  | |  | |  |  | |  | | |  | |  |  | |  | | |  |  |  | |  |  |
|  |  | |  |  | | |  | |  | |  |  | |  | | |  | |  |  | |  | | |  |  |  | |  |  |

| Appendix 3. NAR of nutrients and total energy intake continue | | | | | | | | | | | | |  |  |
| --- | --- | --- | --- | --- | --- | --- | --- | --- | --- | --- | --- | --- | --- | --- |
| **Variables** | **Baseline characteristics** | | | | | | | | | | | | | |
|  | **NAR Vitamin B1** | | | | **NAR Vitamin B2** | | | | **NAR Niacin** | | | | | |
|  | **MEAN** | **±** | **SD** | ***p*-VALUE** | **MEAN** | **±** | **SD** | ***p*-VALUE** | **MEAN** | **±** | **SD** | ***p*-VALUE** | |  |
| **Eating Style** |  |  |  | <.001 |  |  |  | <.001 |  |  |  | <.001 | |  |
| Alone | 1.56 | ± | 0.63 |  | 0.93 | ± | 0.52 |  | 0.84 | ± | 0.38 |  | |  |
| Some together | 1.70 | ± | 0.65 |  | 1.02 | ± | 0.50 |  | 0.94 | # | 0.40 |  | |  |
| Together | 1.72 | ± | 0.63 |  | 0.99 | ± | 0.48 |  | 0.94 | ± | 0.39 |  | |  |
| **Living Arrangement** |  |  |  | 0.024 |  |  |  | 0.030 |  |  |  | 0.012 | |  |
| Living Alone | 1.62 | ± | 0.71 |  | 0.94 | ± | 0.54 |  | 0.88 | ± | 0.44 |  | |  |
| Living with Others | 1.72 | ± | 0.64 |  | 1.01 | ± | 0.49 |  | 0.94 | ± | 0.39 |  | |  |
| **Gender** |  |  |  | <.001 |  |  |  | <.001 |  |  |  | <.001 | |  |
| Male | 1.78 | ± | 0.69 |  | 0.96 | ± | 0.48 |  | 0.97 | ± | 0.42 |  | |  |
| Female | 1.67 | ± | 0.60 |  | 1.04 | ± | 0.49 |  | 0.92 | ± | 0.38 |  | |  |
| **Age Group** |  |  |  | <.001 |  |  |  | <.001 |  |  |  | <.001 | |  |
| 19 - 29 | 1.99 | ± | 0.77 |  | 1.30 | ± | 0.57 |  | 1.14 | ± | 0.47 |  | |  |
| 30 - 39 | 1.91 | ± | 0.71 |  | 1.20 | ± | 0.52 |  | 1.08 | ± | 0.44 |  | |  |
| 40 - 49 | 1.74 | ± | 0.62 |  | 1.04 | ± | 0.48 |  | 0.96 | ± | 0.38 |  | |  |
| 50 - 59 | 1.60 | ± | 0.56 |  | 0.88 | ± | 0.41 |  | 0.87 | ± | 0.35 |  | |  |
| 60 above | 1.48 | ± | 0.51 |  | 0.79 | ± | 0.38 |  | 0.79 | ± | 0.31 |  | |  |
| **Residential Area** |  |  |  | 0.026 |  |  |  | <.001 |  |  |  | 0.001 | |  |
| Urban | 1.72 | ± | 0.65 |  | 1.03 | ± | 0.50 |  | 0.95 | ± | 0.40 |  | |  |
| Rural | 1.67 | ± | 0.62 |  | 0.92 | ± | 0.46 |  | 0.90 | ± | 0.38 |  | |  |
| **BMI** |  |  |  | 0.062 |  |  |  | 0.429 |  |  |  | 0.323 | |  |
| Underweight | 1.66 | ± | 0.65 |  | 1.04 | ± | 0.53 |  | 0.93 | ± | 0.40 |  | |  |
| Normal | 1.70 | ± | 0.63 |  | 1.01 | ± | 0.49 |  | 0.94 | ± | 0.38 |  | |  |
| Overweight | 1.74 | ± | 0.67 |  | 1.00 | ± | 0.49 |  | 0.95 | ± | 0.42 |  | |  |
| **Weight Changes** |  |  |  | <.001 |  |  |  | <.001 |  |  |  | <.001 | |  |
| Same | 1.69 | ± | 0.61 |  | 0.98 | ± | 0.47 |  | 0.93 | ± | 0.38 |  | |  |
| Decreased | 1.64 | ± | 0.64 |  | 0.95 | ± | 0.50 |  | 0.89 | ± | 0.40 |  | |  |
| Increased | 1.80 | ± | 0.70 |  | 1.11 | ± | 0.54 |  | 1.01 | ± | 0.44 |  | |  |
| **Marital Status** |  |  |  | <.001 |  |  |  | <.001 |  |  |  | <.001 | |  |
| Married | 1.69 | ± | 0.61 |  | 0.98 | ± | 0.46 |  | 0.92 | ± | 0.38 |  | |  |
| Once Married | 1.51 | ± | 0.51 |  | 0.85 | ± | 0.41 |  | 0.81 | ± | 0.32 |  | |  |
| Single | 1.95 | ± | 0.79 |  | 1.24 | ± | 0.59 |  | 1.10 | ± | 0.49 |  | |  |
| **Education Level** |  |  |  | <.001 |  |  |  | <.001 |  |  |  | <.001 | |  |
| Primary | 1.44 | ± | 0.51 |  | 0.75 | ± | 0.38 |  | 0.75 | ± | 0.31 |  | |  |
| Secondary | 1.58 | ± | 0.55 |  | 0.86 | ± | 0.39 |  | 0.83 | ± | 0.33 |  | |  |
| Upper Secondary | 1.77 | ± | 0.67 |  | 1.06 | ± | 0.51 |  | 0.97 | ± | 0.41 |  | |  |
| Tertiary | 1.78 | ± | 0.65 |  | 1.08 | ± | 0.49 |  | 1.01 | ± | 0.40 |  | |  |
| **Income Level** |  |  |  | <.001 |  |  |  | <.001 |  |  |  | <.001 | |  |
| Lowest | 1.65 | ± | 0.67 |  | 0.95 | ± | 0.51 |  | 0.89 | ± | 0.41 |  | |  |
| Lower-Middle | 1.67 | ± | 0.62 |  | 0.98 | ± | 0.48 |  | 0.91 | ± | 0.37 |  | |  |
| Upper-Middle | 1.74 | ± | 0.64 |  | 1.02 | ± | 0.48 |  | 0.96 | ± | 0.39 |  | |  |
| Highest | 1.77 | ± | 0.64 |  | 1.06 | ± | 0.50 |  | 1.00 | ± | 0.40 |  | |  |
| **Occupation** |  |  |  | <.001 |  |  |  | <.001 |  |  |  | <.001 | |  |
| White | 1.79 | ± | 0.63 |  | 1.06 | ± | 0.47 |  | 1.01 | ± | 0.39 |  | |  |
| Pink | 1.73 | ± | 0.63 |  | 1.04 | ± | 0.50 |  | 0.95 | ± | 0.39 |  | |  |
| Blue | 1.68 | ± | 0.64 |  | 0.90 | ± | 0.46 |  | 0.89 | ± | 0.38 |  | |  |
| Others | 1.67 | ± | 0.66 |  | 1.03 | ± | 0.52 |  | 0.92 | ± | 0.41 |  | |  |
| **Alcohol Consumption** |  |  |  | 0.118 |  |  |  | <.001 |  |  |  | <.001 | |  |
| Yes | 1.73 | ± | 0.64 |  | 1.02 | ± | 0.49 |  | 0.95 | ± | 0.40 |  | |  |
| No | 1.59 | ± | 0.59 |  | 0.91 | ± | 0.47 |  | 0.86 | ± | 0.37 |  | |  |
| **Cigarettes** |  |  |  | <.001 |  |  |  | <.001 |  |  |  | 0.018 | |  |
| Smoker | 1.79 | ± | 0.72 |  | 1.00 | ± | 0.52 |  | 0.98 | ± | 0.44 |  | |  |
| Once Smoked | 1.72 | ± | 0.64 |  | 0.94 | ± | 0.46 |  | 0.94 | ± | 0.40 |  | |  |
| Non-Smoker | 1.69 | ± | 0.62 |  | 1.03 | ± | 0.49 |  | 0.93 | ± | 0.38 |  | |  |
| **Stress Level** |  |  |  | 0.566 |  |  |  | 0.002 |  |  |  | 0.005 | |  |
| High | 1.76 | ± | 0.69 |  | 1.05 | ± | 0.54 |  | 0.97 | ± | 0.42 |  | |  |
| Medium | 1.71 | ± | 0.63 |  | 1.00 | ± | 0.48 |  | 0.94 | ± | 0.39 |  | |  |
| Low | 1.68 | ± | 0.61 |  | 0.96 | ± | 0.46 |  | 0.91 | ± | 0.38 |  | |  |
| **Nutritional Education** |  |  |  | 0.905 |  |  |  | 0.467 |  |  |  | 0.724 | |  |
| Yes | 1.69 | ± | 0.65 |  | 0.98 | ± | 0.46 |  | 0.93 | ± | 0.40 |  | |  |
| No | 1.71 | ± | 0.64 |  | 1.01 | ± | 0.49 |  | 0.94 | ± | 0.40 |  | |  |
| **Nutrition Supplement Intake (more than2 weeks/year)** |  |  |  | <.001 |  |  |  | <.001 |  |  |  | 0.001 | |  |
| Yes | 1.74 | ± | 0.64 |  | 1.04 | ± | 0.49 |  | 0.96 | ± | 0.40 |  | |  |
| No | 1.69 | ± | 0.64 |  | 0.98 | ± | 0.49 |  | 0.92 | ± | 0.40 |  | |  |
| **Nutritional Fact Usage** |  |  |  | <.001 |  |  |  | <.001 |  |  |  | <.001 | |  |
| Yes | 1.78 | ± | 0.65 |  | 1.12 | ± | 0.50 |  | 1.00 | ± | 0.40 |  | |  |
| No | 1.68 | ± | 0.64 |  | 0.96 | ± | 0.48 |  | 0.92 | ± | 0.39 |  | |  |
| **Total** | 1.71 | ± | 0.64 |  | 1.01 | ± | 0.49 |  | 0.94 | ± | 0.40 |  | |  |
|  |  |  |  |  |  |  |  |  |  |  |  |  | |  |
|  |  |  |  |  |  |  |  |  |  |  |  |  | |  |
|  |  |  |  |  |  |  |  |  |  |  |  |  | |  |
|  |  |  |  |  |  |  |  |  |  |  |  |  | |  |

Appendix 3. NAR of nutrients and total energy intake continue

| **Variables** | **Baseline characteristics** | | | | |  | |  |  |  |
| --- | --- | --- | --- | --- | --- | --- | --- | --- | --- | --- |
|  | **NAR Vitamin C** | | | | |  | |  |  |  |
|  | **MEAN** | **±** | **SD** | ***p*-VALUE** |  | |  | |  |  |
| **Eating Style** |  |  |  | 0.093 |  | |  | |  |  |
| Alone | 1.11 | ± | 0.89 |  |  | |  | |  |  |
| Some together | 1.25 | ± | 0.89 |  |  | |  | |  |  |
| Together | 1.23 | ± | 0.82 |  |  | |  | |  |  |
| **Living Arrangement** |  |  |  | 0.007 |  | |  | |  |  |
| Living Alone | 1.08 | ± | 0.84 |  |  | |  | |  |  |
| Living with Others | 1.24 | ± | 0.85 |  |  | |  | |  |  |
| **Gender** |  |  |  | <.001 |  | |  | |  |  |
| Male | 1.10 | ± | 0.73 |  |  | |  | |  |  |
| Female | 1.33 | ± | 0.91 |  |  | |  | |  |  |
| **Age Group** |  |  |  | 0.210 |  | |  | |  |  |
| 19 29 | 1.21 | ± | 0.77 |  |  | |  | |  |  |
| 30 - 39 | 1.23 | ± | 0.77 |  |  | |  | |  |  |
| 40 - 49 | 1.19 | ± | 0.82 |  |  | |  | |  |  |
| 50 - 59 | 1.27 | ± | 0.89 |  |  | |  | |  |  |
| 60 above | 1.24 | ± | 0.92 |  |  | |  | |  |  |
| **Residential Area** |  |  |  | 0.015 |  | |  | |  |  |
| Urban | 1.24 | ± | 0.84 |  |  | |  | |  |  |
| Rural | 1.17 | ± | 0.88 |  |  | |  | |  |  |
| **BMI** |  |  |  | 0.158 |  | |  | |  |  |
| Underweight | 1.13 | ± | 0.76 |  |  | |  | |  |  |
| Normal | 1.24 | ± | 0.84 |  |  | |  | |  |  |
| Overweight | 1.21 | ± | 0.86 |  |  | |  | |  |  |
| **Weight Changes** |  |  |  | 0.011 |  | |  | |  |  |
| Same | 1.23 | ± | 0.84 |  |  | |  | |  |  |
| Decreased | 1.16 | ± | 0.85 |  |  | |  | |  |  |
| Increased | 1.28 | ± | 0.87 |  |  | |  | |  |  |
| **Marital Status** |  |  |  | <.001 |  | |  | |  |  |
| Married | 1.25 | ± | 0.85 |  |  | |  | |  |  |
| Once Married | 1.06 | ± | 0.79 |  |  | |  | |  |  |
| Single | 1.20 | ± | 0.84 |  |  | |  | |  |  |
| **Education Level** |  |  |  | <.001 |  | |  | |  |  |
| Primary | 1.05 | ± | 0.82 |  |  | |  | |  |  |
| Secondary | 1.21 | ± | 0.89 |  |  | |  | |  |  |
| Upper Secondary | 1.25 | ± | 0.82 |  |  | |  | |  |  |
| Tertiary | 1.28 | ± | 0.86 |  |  | |  | |  |  |
| **Income Level** |  |  |  | <.001 |  | |  | |  |  |
| Lowest | 1.09 | ± | 0.79 |  |  | |  | |  |  |
| Lower-Middle | 1.13 | ± | 0.76 |  |  | |  | |  |  |
| Upper-Middle | 1.26 | ± | 0.83 |  |  | |  | |  |  |
| Highest | 1.41 | ± | 0.94 |  |  | |  | |  |  |
| **Occupation** |  |  |  | <.001 |  | |  | |  |  |
| White | 1.26 | ± | 0.83 |  |  | |  | |  |  |
| Pink | 1.20 | ± | 0.81 |  |  | |  | |  |  |
| Blue | 1.09 | ± | 0.75 |  |  | |  | |  |  |
| Others | 1.33 | ± | 0.92 |  |  | |  | |  |  |
| **Alcohol Consumption** |  |  |  | 0.013 |  | |  | |  |  |
| Yes | 1.22 | ± | 0.84 |  |  | |  | |  |  |
| No | 1.32 | ± | 0.91 |  |  | |  | |  |  |
| **Cigarettes** |  |  |  | <.001 |  | |  | |  |  |
| Smoker | 0.99 | ± | 0.70 |  |  | |  | |  |  |
| Once Smoked | 1.15 | ± | 0.74 |  |  | |  | |  |  |
| Non-Smoker | 1.31 | ± | 0.89 |  |  | |  | |  |  |
| **Stress Level** |  |  |  | 0.257 |  | |  | |  |  |
| High | 1.20 | ± | 0.89 |  |  | |  | |  |  |
| Medium | 1.23 | ± | 0.83 |  |  | |  | |  |  |
| Low | 1.26 | ± | 0.84 |  |  | |  | |  |  |
| **Nutritional Education** |  |  |  | 0.197 |  | |  | |  |  |
| Yes | 1.31 | ± | 0.85 |  |  | |  | |  |  |
| No | 1.23 | ± | 0.85 |  |  | |  | |  |  |
| **Nutrition Supplement Intake (more than2 weeks/year)** |  |  |  | <.001 |  | |  | |  |  |
| Yes | 1.30 | ± | 0.85 |  |  | |  | |  |  |
| No | 1.17 | ± | 0.84 |  |  | |  | |  |  |
| **Nutritional Fact Usage** |  |  |  | <.001 |  | |  | |  |  |
| Yes | 1.39 | ± | 0.92 |  |  | |  | |  |  |
| No | 1.17 | ± | 0.81 |  |  | |  | |  |  |
| **Total** | 1.23 | ± | 0.85 |  |  | |  | |  |  |
