# Supplementary material for: Association between eating behaviour and diet quality: eating alone vs. eating with others
Source: Nutr J. 2018 Dec 19;17:117. doi: 10.1186/s12937-018-0424-0 (PMC6299993; doi:10.1186/s12937-018-0424-0)
Supplement: Supplementary file 2 — Appendix 4. Association between variables and MAR by cutoff value at 0.5 Appendix 5. Results of adjusted and unadjusted model related to socioeconomic factors Appendix 6. Interaction between variables. (DOCX 24.3 kb) [file 12937_2018_424_MOESM2_ESM.docx]

Appendix 4. Association between variables and MAR by cutoff value at 0.5

| **Variables** | **MAR** | | | | | | | |
| --- | --- | --- | --- | --- | --- | --- | --- | --- |
|  | **Male** | | | |  | **Female** |  |  |
|  | **OR** | **95% CI** | | | **OR** | **95% CI** | | |
| **Eating Behaviour** |  |  |  |  |  |  |  |  |
| Alone | 0.52 | 0.27 | - | 1.01 | 0.84 | 0.54 | - | 1.31 |
| Some Together | 1.07 | 0.66 | - | 1.74 | 0.98 | 0.73 | - | 1.33 |
| Together | 1.00 |  | - |  | 1.00 |  | - |  |
| **Living Arrangement** |  |  |  |  |  |  |  |  |
| Living Alone | 1.40 | 0.65 | - | 3.02 | 0.79 | 0.48 | - | 1.31 |
| Living with Others | 1.00 |  | - |  | 1.00 |  | - |  |
| **Age Group** |  |  |  |  |  |  |  |  |
| Age<30 | 0.68 | 0.25 | - | 1.83 | 0.56 | 0.31 | - | 1.04 |
| Age<40 | 0.37 | 0.14 | - | 0.95 | 0.46 | 0.24 | - | 0.87 |
| Age<50 | 0.31 | 0.11 | - | 0.85 | 0.50 | 0.25 | - | 0.99 |
| Age<60 | 0.25 | 0.08 | - | 0.74 | 0.51 | 0.24 | - | 1.09 |
| Age<70 | 1.00 |  | - |  | 1.00 |  | - |  |
| **Residential Area** |  |  |  |  |  |  |  |  |
| Urban | 0.93 | 0.54 | - | 1.60 | 1.01 | 0.69 | - | 1.47 |
| Rural | 1.00 |  | - |  | 1.00 |  | - |  |
| **BMI** |  |  |  |  |  |  |  |  |
| Underweight | 0.73 | 0.23 | - | 2.27 | 0.77 | 0.44 | - | 1.36 |
| Overweight | 0.91 | 0.58 | - | 1.42 | 1.00 | 0.73 | - | 1.37 |
| Normal | 1.00 |  | - |  | 1.00 |  | - |  |
| **Weight Changes** |  |  |  |  |  |  |  |  |
| Same | 1.00 |  | - |  | 1.00 |  | - |  |
| Decreased | 0.54 | 0.32 | - | 0.92 | 0.83 | 0.56 | - | 1.24 |
| Increased | 0.96 | 0.52 | - | 1.74 | 0.92 | 0.67 | - | 1.27 |
| **Marital Status** |  |  |  |  |  |  |  |  |
| Married | 2.03 | 0.91 | - | 4.54 | 2.55 | 1.48 | - | 4.37 |
| Separated | 0.92 | 0.38 | - | 2.26 | 1.74 | 0.94 | - | 3.23 |
| Single | 1.00 |  | - |  | 1.00 |  | - |  |
| **Education Level** |  |  |  |  |  |  |  |  |
| Primary | 0.27 | 0.12 | - | 0.60 | 0.39 | 0.23 | - | 0.68 |
| Secondary | 0.89 | 0.34 | - | 2.33 | 0.44 | 0.26 | - | 0.75 |
| Upper Secondary | 0.47 | 0.25 | - | 0.88 | 0.73 | 0.49 | - | 1.08 |
| Tertiary | 1.00 |  | - |  | 1.00 |  | - |  |
| **Income Level** |  |  |  |  |  |  |  |  |
| Lowest | 0.35 | 0.16 | - | 0.81 | 0.25 | 0.15 | - | 0.43 |
| Lower-Middle | 0.47 | 0.21 | - | 1.06 | 0.39 | 0.23 | - | 0.66 |
| Upper-Middle | 0.49 | 0.21 | - | 1.16 | 0.47 | 0.27 | - | 0.82 |
| Highest | 1.00 |  | - |  | 1.00 |  | - |  |
| **Occupation** |  |  |  |  |  |  |  |  |
| White | 1.75 | 0.81 | - | 3.79 | 1.30 | 0.84 | - | 2.00 |
| Pink | 3.17 | 1.17 | - | 8.56 | 1.36 | 0.92 | - | 2.01 |
| Blue | 1.71 | 0.98 | - | 2.97 | 1.71 | 1.13 | - | 2.58 |
| Others | 1.00 |  | - |  | 1.00 |  | - |  |
| **Alcohol Consumption** |  |  |  |  |  |  |  |  |
| Yes | 1.09 | 0.37 | - | 3.21 | 1.17 | 0.78 | - | 1.74 |
| No | 1.00 |  | - |  | 1.00 |  | - |  |
| **Cigarettes** |  |  |  |  |  |  |  |  |
| Smoker | 0.53 | 0.28 | - | 1.01 | 1.00 | 0.58 | - | 1.73 |
| Ex-Smoker | 0.96 | 0.47 | - | 1.95 | 0.50 | 0.32 | - | 0.79 |
| Non-Smoker | 1.00 |  | - |  | 1.00 |  | - |  |
| **Stress Level** |  |  |  |  |  |  |  |  |
| High | 1.03 | 0.52 | - | 2.01 | 0.76 | 0.48 | - | 1.22 |
| Medium | 1.21 | 0.66 | - | 2.20 | 0.89 | 0.58 | - | 1.37 |
| Low | 1.00 |  | - |  | 1.00 |  | - |  |
| **Nutritional Education** |  |  |  |  |  |  |  |  |
| No | 1.29 | 0.42 | - | 3.95 | 0.49 | 0.18 | - | 1.35 |
| Yes | 1.00 |  | - |  | 1.00 |  | - |  |
| **Nutrition Supplement Intake (more than2 weeks/year)** | | | | | |  |  |  |
| No | 0.39 | 0.22 | - | 0.68 | 0.65 | 0.49 | - | 0.86 |
| Yes | 1.00 |  | - |  | 1.00 |  | - |  |
| **Nutritional Fact Usage** |  |  |  |  |  |  |  |  |
| No | 0.49 | 0.22 | - | 1.09 | 0.61 | 0.44 | - | 0.86 |
| Yes | 1.00 |  | - |  | 1.00 |  | - |  |

Appendix 5. Results of adjusted and unadjusted model related to socioeconomic factors

| **Variables** | | | **MAR** | | | | | | | | |
| --- | --- | --- | --- | --- | --- | --- | --- | --- | --- | --- | --- |
|  |  |  |  | **Male** |  |  | | **Female** | | |  |
|  |  |  | **β** | **S.E** | | | ***p*-VALUE** | **β** | **S.E** | ***p*-VALUE** | |
| **Model 1** | **Eating Behaviour** | | | | | | | | | | |
|  | Alone | | -0.114 | 0.035 | | | 0.001 | -0.076 | 0.024 | 0.002 | |
|  | Some-with others | | -0.002 | 0.018 | | | 0.923 | -0.010 | 0.014 | 0.474 | |
|  | With others | | Ref. |  | | |  | Ref. |  |  | |
| **Model 2** | **Eating Behaviour** | |  |  | | |  |  |  |  | |
|  | Alone | | -0.117 | 0.035 | | | 0.001 | -0.068 | 0.024 | 0.006 | |
|  | Some-with others | | -0.003 | 0.018 | | | 0.887 | -0.007 | 0.014 | 0.593 | |
|  | With others | | Ref. |  | | |  | Ref. |  |  | |
| **Model 3** | **Eating Behaviour** | |  |  | | |  |  |  |  | |
|  | Alone | | -0.115 | 0.035 | | | 0.001 | -0.068 | 0.024 | 0.005 | |
|  | Some-with others | | -0.002 | 0.018 | | | 0.932 | -0.010 | 0.014 | 0.464 | |
|  | With others | | Ref. |  | | |  | Ref. |  |  | |
| **Model 4** | **Eating Behaviour** | |  |  | | |  |  |  |  | |
|  | Alone | | -0.123 | 0.035 | | | 0.000 | -0.075 | 0.025 | 0.002 | |
|  | Some-with others | | -0.003 | 0.018 | | | 0.883 | -0.007 | 0.014 | 0.617 | |
|  | With others | | Ref. |  | | |  | Ref. |  |  | |
| **Model 5** | **Eating Behaviour** | |  |  | | |  |  |  |  | |
|  | Alone | | -0.118 | 0.035 | | | 0.001 | -0.074 | 0.024 | 0.003 | |
|  | Some-with others | | -0.002 | 0.018 | | | 0.930 | -0.010 | 0.014 | 0.474 | |
|  | With others | | Ref. |  | | |  | Ref. |  |  | |
| **Model 6** | **Eating Behaviour** | |  |  | | |  |  |  |  | |
|  | Alone | | -0.125 | 0.035 | | | 0.000 | -0.067 | 0.024 | 0.006 | |
|  | Some-with others | | -0.004 | 0.018 | | | 0.844 | -0.009 | 0.014 | 0.531 | |
|  | With others | | Ref. |  | | |  | Ref. |  |  | |
| **Model 7** | **Eating Behaviour** | |  |  | | |  |  |  |  | |
|  | Alone | | -0.131 | 0.035 | | | 0.000 | -0.074 | 0.024 | 0.002 | |
|  | Some-with others | | -0.004 | 0.018 | | | 0.824 | -0.008 | 0.014 | 0.555 | |
|  | With others | | Ref. |  | | |  | Ref. |  |  | |
|  |  | |  |  | | |  |  |  |  | |
|  |  | |  |  | | |  |  |  |  | |
| Model 1 | | Fully adjusted except education level | | | | | | | | | |
| Model 2 | | Fully adjusted except income level | | | | | | | | | |
| Model 3 | | Fully adjusted except occupation | | | | | | | | | |
| Model 4 | | Fully adjusted except education level and income level | | | | | | | | | |
| Model 5 | | Fully adjusted except education level and occupation | | | | | | | | | |
| Model 6 | | Fully adjusted except income level and occupation | | | | | | | | | |
| Model 7 | | Fully adjusted except education level, income level, and occupation | | | | | | | | | |

Appendix 6. Interaction between variables

|  | **Variable** | **Estimate** | **S.D** | **P-value** |
| --- | --- | --- | --- | --- |
| Male | edu*EAT | 1.080 | 0.120 | 0.044 |
| Female | edu*EAT | 4.094 | 0.455 | <.0001 |
|  |  |  |  |  |
| Male | incm*EAT | 1.690 | 0.188 | 0.001 |
| Female | incm*EAT | 2.710 | 0.301 | <.0001 |
|  |  |  |  |  |
| Male | occp*EAT | 1.119 | 0.124 | 0.036 |
| Female | occp*EAT | 1.708 | 0.190 | 0.008 |

EAT: eating behaviour, edu: education, incm: income, occp: occupation
